# Encountering the clinical complexity of type II Peters anomaly management approaches: a case report

**DOI:** 10.11604/pamj.2024.49.47.44754

**Published:** 2024-10-22

**Authors:** Devi Sarah Intan Permatasari, Dicky Hermawan, Rozalina Loebis

**Affiliations:** 1 Department of Ophthalmology, Faculty of Medicine Universitas Airlangga, Dr. Soetomo General Academic Hospital, Surabaya, Indonesia

**Keywords:** Anterior segment dysgenesis, Peters anomaly, keratolenticular adhesion, lens aspiration, case report

## Abstract

Anterior segment dysgenesis exerts its influence on a diverse array of ocular structures, encompassing the cornea, iris, ciliary body, anterior chamber and lens. We present a 20-month-old boy with bilateral corneal opacity. The visual acuity (VA) was 6/480 in both eyes. Upon examination, we found bilateral central corneal opacity with keratolenticular adhesions, anterior lens dislocation and opacification, aniridia. The clinical findings indicate diagnosis features of type II Peters anomaly (PA). Lens aspiration combined with adhesiolysis on the left eye (LE) was performed to address cataract-induced visual axis obstruction and prevent corneal decompensation from keratolenticular adhesions. We contemplated on prioritizing surgery for the LE initially due to the less severe corneal opacity compared to the right eye (RE). Further evaluations are required to determine the visual enhancement and the necessity of additional procedures. The management of type II PA proved to be a challenging experience. Cautious manipulation and extensive counseling can prevent further corneal decompensation.

## Introduction

The term anterior segment developmental disorders (ASDA) refers to ocular developmental anomalies related with this anatomical portion of the human eye organ known as anterior segment dysgenesis (ASD). ASD diseases can impact the cornea, iris, ciliary body, anterior chamber and lens, exhibiting a broad range of clinical symptoms. Many developmental abnormalities, including Peters anomaly (PA), Axenfeld-Rieger syndrome (ARS) and aniridia, are brought on by improper interaction [1].

PA is an uncommon variation of congenital anterior segment dysgenesis. The main characteristics are lenticulo-corneal or irido-corneal synechiae and varying degrees of central corneal opacity, characterized by a central corneal opacity with defects in the corneal endothelium, Descemet's membrane, and posterior stroma; adhesion of the iris and cornea are also present [2]. An uncommon ailment known as congenital corneal opacification affects 6 out of every 100,000 infants worldwide [3]. Even though they are rare, corneal opacities can have a catastrophic impact on a child's long-term vision, particularly if they are not identified and treated right away. There are several etiologic causes for congenital cloudy cornea, including metabolic, developmental, genetic, and idiopathic. There are still no standard treatment approval established yet, however, various surgical approaches have been studied through the years, the approachements are penetrating keratoplasty, cataract aspiration or lensectomy, optical iridectomy, selective endothelial removal, trabeculectomy and implant of glaucoma draining devices [4-6].

The management of congenital corneal opacities in infants is challenging, and the approach must be tailored to the specific cause and child. This study aims to present ocular manifestation of the anterior segment dysgenesis, summarize treatment options to preserve the patient's visual acuity, prevent further complications, and describe the importance of improving care coordination among interprofessional to improve outcomes. We report a case of a 20-month-old male with bilateral corneal opacification and keratolenticular adhesion present since birth, diagnosed with Type II Peters anomaly.

## Patient and observation

**Patient information:** a 20-month-old boy was administered with a chief complaint of whitish appearance in both eyes which occurred since the patient's birth. The patient was also said to be having spontaneous movements in both eyes. Redness in both eyes or the surrounding areas, family history of similar condition, systemic disease, and past illness during gestation were denied.

**Figure 1:**
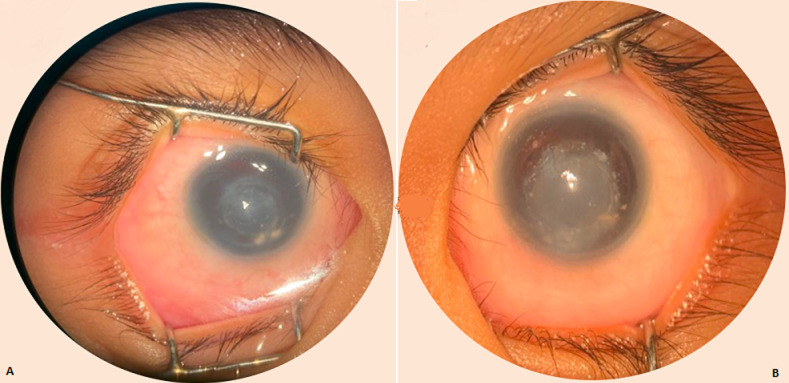
anterior segment examination of both eyes: A) right eye; B) left eye

**Clinical findings:** ophthalmologic examination performed in patient found that the binocular visual acuity (VA) was 6/480 (Lea Grating). Through clinical examination, nystagmus and bilateral corneal opacity were found in both eyes. The comprehensive examinations performed by pediatrician showed that the patient suffered no systemic abnormality or disease. Central corneal opacity accompanied by deposited material was present bilaterally, along with keratolenticular adhesions in both eyes. The peripheral area of cornea remains clear. There were no Haab's striae and vascularization on the cornea in both eyes. Anterior chamber was difficult to evaluate. Aniridia was found in both eyes. Pupils in the RE and LE were roundly shaped, 8 mm and 7 mm in diameter respectively, and the light reflexes were difficult to evaluate. Posterior segment examination revealed that positive fundus reflex was seen on the posterior pole, with normal detail configuration (Figure 1).

**Diagnostic assessment:** we performed ophthalmologic ultrasound examination and found that both of the patient's eyes are within normal range. The examination under anesthesia (EUA) revealed that the intraocular pressure (IOP) in the right eye (RE) and left eye (LE) were 17.3 mmHg and 14.6 mmHg respectively.

**Diagnosis:** type II Peters anomaly.

**Figure 2:**
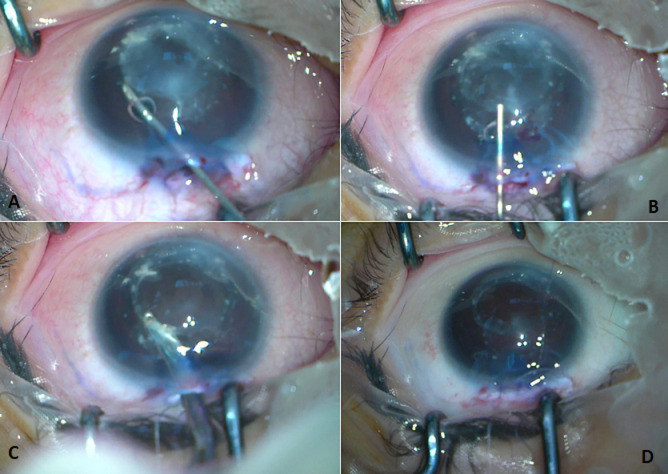
(A, B) intraoperative procedure; C) adhesiolysis was mechanically conducted using a cystotome and viscoelastic injection; D) lens material aspiration

**Figure 3:**
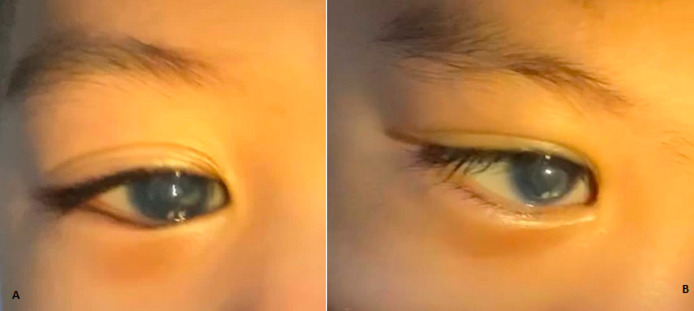
A) exenteration and surgical debridement of the frontal and zygoma subcutaneous abscess; B) opacity was notably reduced one day after the procedure

**Therapeutic interventions:** we performed lens aspiration combined with adhesiolysis on the patient's LE. The adhesiolysis procedure was done mechanically with cystotome and viscoelastic injection (Figure 2). There were no intra-operational complications reported. The patient was given topical antibiotic, corticosteroid, and anti-glaucoma medication post-operatively. The post-operative course showed no complication and there was a notable reduction in corneal opacity (Figure 3).

**Follow-up and outcome of interventions:** we conducted the post-operative follow-up assessment on patient. Post-operatively, the patient showed no signs of complications or condition worsening regarding the initial complaints and following the procedure.

**Informed consent:** parents were acknowledged about the case reported and agreed that the case would be published for the benefit of people and medicine.

## Discussion

Anterior segment dysgenesis (ASD) is a rare group of disorders that lead to corneal opacity due to anterior segment dysgenesis during developmental period. The following terms can be easily remembered by using the acronym STUMPED: sclerocornea, ulcers (infection), metabolic disorders (such as mucopolysaccharidosis), tears in the Descemet membrane (usually caused by forceps trauma or congenital glaucoma), Peters anomaly, edema (such as congenital hereditary endothelial dystrophy (CHED), posterior polymorphous dystrophy, congenital hereditary stromal dystrophy (CHSD), glaucoma), and dermoid [7]. In this case, 20-month-old patient came to ophthalmology clinic complained of whitish appearance on both eyes since birth. PA is described as a central corneal opacity with corneal endothelium defects, Descemet's membrane, and posterior stroma. Adhesion of the lens and cornea may also be present. Visual acuity was 6/480 by Lea Grating (binocular). Any anomalies or adjustments to the corneal shape may impact a patient's refractive functions. Anisometropia can nevertheless cause a major visual deficit even in cases when corneal opacity is peripheral and indirectly in the visual axis. The opacity blocking the visual axis can cause visual deprivation amblyopia [8]. The IOP of the RE was 17.3 mmHg and in the LE was 14.6 mmHg. Generally, anterior segment anomalies are associated with an approximate 50% risk of glaucoma. In addition, 50%-70% infants with PA also have glaucoma. Glaucoma can arise due to the IOP level increase which caused by aqueous humor flow disturbance related to structural dysgenesis in the anterior segment. Congenital sensory nystagmus was present in this case as secondary to an early-onset, bilateral abnormality of the pregeniculate afferent visual pathway. In this patient, corneal opacity obstructs the visual axis resulting inadequate retinal image formation. This condition interferes the fixation reflex so that nystagmus can occur. Corneal opacity showed central corneal opacity and remain clear on peripheral area and there was keratolenticular adhesion present in both eyes. Corneal leukoma with iridocorneal adhesion is classified as type I Peters anomaly, and corneal leukoma with cataract and keratolenticular adhesion is classified as type II Peters anomaly [6].

PA is typified by anterior segment dysgenesis, which explains the most severe kind of anomaly in "corneal congenital opacities." Regarding management, there is no one-size-fits-all method of curing the condition, and the best time to have surgery is still up for debate. While its efficacy in type I PA is undeniable, Penetrating keratoplasty (PKP) faces an uphill battle in type II. Parekh *et al*. stated that the delicate dance between dissecting keratolenticular adhesions and preserving corneal integrity poses a high risk of further damage and graft rejection. Additionally, managing the cataract concurrently adds complexity and can compromise graft clarity. Despite these challenges, PKP remains a viable option for certain cases, particularly those with a clear zone in the peripheral cornea and minimal adhesions [8]. In contrast, patients with type II anomalies require a range of procedures such as adhesiolysis, lens aspiration, vitrectomy, and glaucoma surgery (trabeculectomy, glaucoma drainage device, laser cycloablation), which worsens the outcome of a corneal transplant. Patients with type I anomalies should have PKP or optical-sector iridectomy within the first year of life. Although there isn't a single "gold-standard" method, most authors suggest PKP. Zaidman assessed the visual prognosis following corneal transplantation for twenty-four individuals with type I Peters abnormality. During a 78.9-month follow-up, he discovered that 90% of children over the age of three had clear corneas, and 83% of children who had surgery in their first year had done so. Fifty percent of the cases were treated for glaucoma, and 17 percent of the cases had graft rejection [6].

In a Korean study based on 19 PKP performed in children over 1 year with Peters abnormality, graft failure ranges from 30% at 1 year, 39% at 3 years, 70% at 5 years and 77% at 10 years. A second retrospective case series that looked at the outcomes of kids with PK identified the need for numerous surgical procedures and the onset of glaucoma as separate causes of graft failure. Pediatric PKP is a complicated operation that involves regular exams under general anesthesia and chronic topical steroid administration, which can have long-term consequences in addition to a high chance of graft failure [9]. If there's a clear zone in the cornea, an alternative surgery called optical iridectomy was suggested to get a free visual axis. The biggest series of 29 optical iridectomies for type I Peters anomaly in children under 8 years old was published by Oriel Spierer (2018), who showed that the treatment was successful in improving visual function in 72.4% of patients over a 42.8-month period, especially in bilateral cases. This less invasive technique offers a glimmer of hope by creating a clear visual pathway through partial iridectomy, potentially improving visual acuity without full-blown corneal transplantation. However, its effectiveness diminishes in cases with extensive corneal opacity or significant adhesions with potential glare and cosmetic concerns require careful consideration. Optical iridectomy serves as a valuable tool in the armamentarium, particularly for unilateral cases or when PKP risks outweigh potential benefits [9]. More research is required to determine the efficacy of optical iridectomy in unilateral instances. In a case of type I PA, Soh *et al*. reported a novel method that involved selective endothelium ablation while maintaining intact DM. The patient's visual acuity increased to 20/30 without requiring another corneal transplant [5]. The surgical method is more challenging in type II anomalies due to corneal opacity and kerato-lenticular adhesions. To the best of our knowledge, type II PA surgical alternatives are lacking in the literature. Beyond established options, the future holds promise for more targeted and effective approaches. According to a study conducted by Ramappa *et al*. the use of Ahmed glaucoma valve (AGV) implantation in the management of glaucoma in Peters anomaly has shown promising results. AGV implantation can be used as an alternative to trabeculectomy in selected cases. Selective endothelial removal is a novel technique that preserves Descemet's membrane and replaces only the dysfunctional corneal endothelium, potentially reducing the risks associated with full-thickness PKP and offering improved long-term outcomes [7,10].

Navigating the complex landscape of type II PA necessitates a patient-centered approach, meticulously tailoring treatment strategies to each individual's unique presentation. Key factors to consider include severity of corneal opacity and adhesions, presence of glaucoma, and patient age and overall health. Extensive involvement may necessitate a staged approach, starting with less invasive interventions like optical iridectomy and progressing to PKP only if necessary [1]. Early and aggressive management of glaucoma is crucial to prevent irreversible vision loss, requiring close collaboration with glaucoma specialists. Younger patients and those with comorbidities demand a cautious approach, carefully weighing the risks and benefits of each intervention.

## Conclusion

The management of type II PA proved to be a challenging experience. Anterior segment dysgenesis has various ocular manifestations that can indicate to another systemic or genetic conditions. Early diagnostic and prompt treatment are required to establish the best clinical outcomes and prognosis. Collaboration with related divisions and departments is needed to make a comprehensive work up. No surgical intervention is superior to another. The selection is based on each case and possible complications can occur following the surgery. Cautious handling techniques have the potential to mitigate the risk of subsequent corneal decompensation. Furthermore, comprehensive counseling should be provided to parents concerning potential complications and prognosis.
